# Astaxanthin from Shrimp By-Products Enhances Oxidative Stability of Lard During Storage

**DOI:** 10.3390/foods14152585

**Published:** 2025-07-23

**Authors:** Olga Draghici

**Affiliations:** Department of Agricultural Sciences and Food Engineering, Lucian Blaga University of Sibiu, 7–9 Dr. Ion Ratiu Street, 550024 Sibiu, Romania; olga.draghici@ulbsibiu.ro; Tel.: +40-2-6923-6441

**Keywords:** astaxanthin, lard, lipid oxidation, thermal stability

## Abstract

Previous research has primarily focused on the antioxidant effect of astaxanthin (AX) in various vegetable oils, with limited attention given to its behavior in lard. This study aimed to evaluate the degradation of AX during lard storage and to assess the physicochemical changes occurring in lard containing different AX concentrations over time. The variation in AX concentration was monitored using spectrophotometric analysis. To characterize the changes in lard, both thermal and chemical methods were employed: thermal analysis was used to determine the onset oxidation temperature (To) and activation energy (Ea), while chemical methods included peroxide value (PV) and thiobarbituric acid reactive substance (TBA) assays. Optimization of AX concentration and temporal evaluation of its antioxidant effect were performed using Response Surface Methodology (RSM). The results indicated a significant degradation of AX after 30 days of storage. An AX concentration of approximately 3 mg/g was identified as optimal, as it provided the highest thermal stability and the lowest levels of oxidation markers, offering a well-balanced compromise between technological performance and preservative effectiveness in lard during storage. Additionally, the color of the lard was found to be more strongly influenced by the presence of AX itself rather than by its specific concentration.

## 1. Introduction

The scientific literature provides extensive evidence supporting the efficacy of various antioxidant compounds, both synthetic and natural, in enhancing the oxidative stability of edible oils. In recent years, there has been an increasing emphasis on identifying natural alternatives as safer substitutes for synthetic antioxidants [1]. In contrast, the oxidative degradation of animal fats in the presence of antioxidants has been addressed to a significantly lesser extent in scientific investigations [2,3].

Lard, a commonly used animal fat, has been reported to contain considerable amounts of monounsaturated (MUFAs) and polyunsaturated fatty acids (PUFAs), the composition of which is influenced by pig breed and dietary intake [4]. This lipid profile makes lard particularly susceptible to oxidative deterioration, thereby necessitating the use of protective strategies against oxidation.

Natural antioxidants have recently gained traction as promising alternatives to synthetic compounds in protecting edible fats from oxidative degradation [5,6]. Among them, astaxanthin (AX), a carotenoid with potent antioxidant activity, has attracted significant attention. While AX is predominantly employed in aquaculture to enhance fish pigmentation [7], it also offers well-documented health benefits [8] and has found applications in the food [5,9], nutraceutical [10], and cosmetic sectors [11]. Astaxanthin used as an antioxidant may be obtained from both natural and synthetic sources.

Shrimp processing by-products represent a rich and sustainable source of natural astaxanthin, present in both free and fatty acid-esterified forms [12]. The valorization of such food by-products has become increasingly relevant in light of current market trends toward sustainability, waste reduction, and efficient resource utilization through innovative solutions [13].

Conventional extraction methods of astaxanthin rely on its solubility in organic solvents or vegetable oils but are often limited in terms of efficiency and environmental impact. In contrast, emerging techniques such as ultrasound-assisted extraction and pulsed electric field treatment offer more effective and eco-friendly alternatives. Among these, high-pressure processing is particularly noteworthy due to its superior extraction yields, enhanced product quality, and reduced processing time, positioning it as an advanced alternative for industrial applications [14].

Several studies have confirmed the ability of AX to enhance the oxidative stability of meat products [6]. However, to date, no research has been identified that specifically addresses the antioxidant effect of AX on lard, highlighting the novelty and relevance of the present study.

It has also been reported that the concentration of AX in lipid-based food systems can decrease over time to varying extents, depending on environmental and storage conditions. This decline is primarily attributed to oxidation processes and the formation of degradation products such as apoastaxanthins [1,15].

The aim of this research was to evaluate the antioxidant effect of AX obtained from shrimp by-products on the oxidative stability of lard over time, by monitoring key oxidation indicators. In this context, different methods were compared for tracking the degradation of AX over time. Subsequently, the AX concentration was optimized and the antioxidant effect was assessed over time using the Response Surface Methodology. The degradation of AX in lard during storage was also modeled.

## 2. Materials and Methods

### 2.1. Materials

Commercially purchased refrigerated shrimp were manually peeled, and the resulting by-products (including heads, shells, tails, and legs) were homogenized using a blender (Philips HR1372/90, IKA Digital, Staufen, Germany) [16]. Subcutaneous adipose tissue was sourced from a local retail market and processed by dry rendering [17].

The rendered fat was then separated by decantation and cooled to 50 °C. The following physicochemical properties of the obtained lard were determined: melting point—33.12 °C; iodine value—48.7 g I_2_/100 g fat; peroxide value—2.42 meq O_2_/kg.

### 2.2. AX Extraction and Preparation of the Lard–AX System

**Figure 1:**
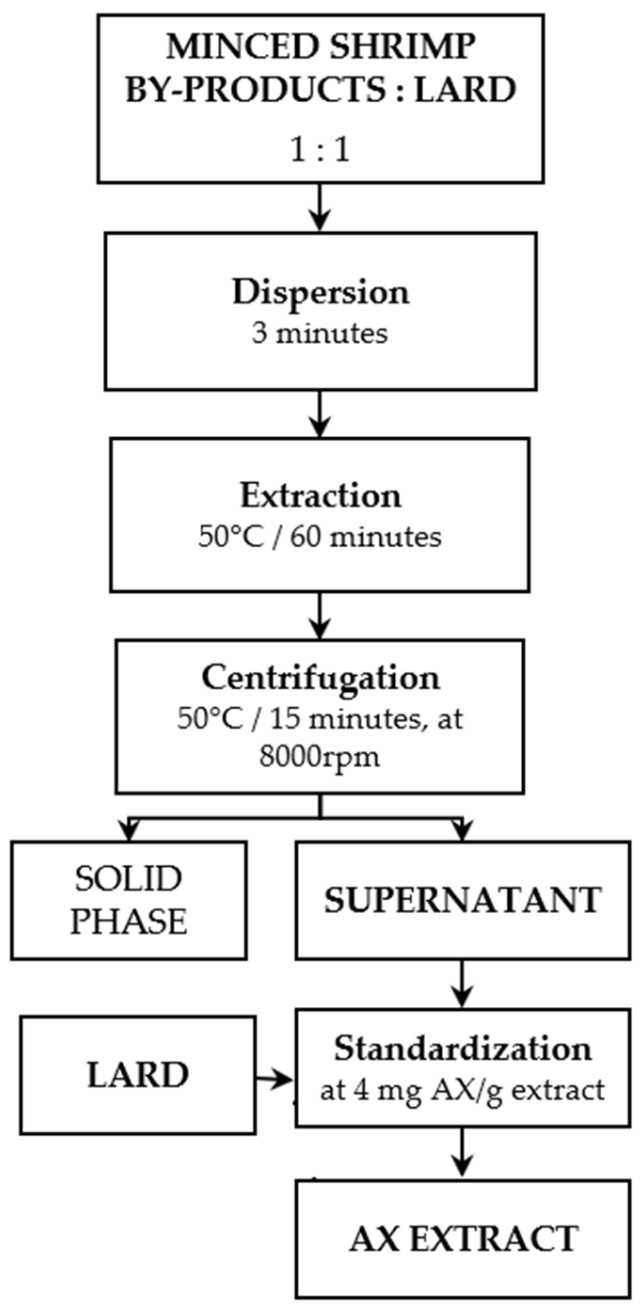
Extracting astaxanthin from shrimp by-products using lard.

Astaxanthin (AX) was extracted from shrimp by-products following the method described by Pu et al. and Sachindra et al. [18,19], with several modifications, as illustrated in Figure 1. The minced shrimp by-products were mixed with lard in a 1:1 ratio and homogenized for 3 min using an ULTRA-TURRAX homogenizer (IKA T 25 Digital, Staufen, Germany). The extraction was subsequently carried out at 50 °C for 60 min using a magnetic stirrer (Phönix Instrument RSM-10HS, Phönix Instrument, Garbsen, Germany). The AX-rich lipid phase was separated by centrifugation (Nuve NF 1200R, Nuve, Ankara, Turkey) at 50 °C for 15 min at 8000 rpm. The AX concentration in the supernatant was determined spectrophotometrically using the method described by Takeungwongtrakul and Benjakul [20]. In brief, 30 mg of the supernatant was mixed with 20 mL of petroleum ether by shaking for 5 min, then left to stand for 30 min. The absorbance was measured using a spectrophotometer at 468 nm. The same procedure was followed with varying amounts of pure astaxanthin to obtain the calibration curve.

After that the supernatant was diluted with lard to a final AX concentration of 4 mg/g.

Previous studies have shown that cooked shrimp shells contain only about 2.10% lipids, as reported by Dave et al. [16], with comparable values observed by Gómez-Estaca et al. [21]. Among the liposoluble antioxidants recoverable through the aforementioned extraction method is astaxanthin (AX), which occurs in both free and esterified forms. Astaxanthin is the primary carotenoid found in the by-products resulting from shrimp processing, being present in both its free and esterified forms. Previous studies have shown that the by-products from *Litopenaeus vannamei* processing exhibit a predominant proportion of astaxanthin diesters (43%), followed by monoesters (41%) and free astaxanthin (16%).

Since the present study aims to explore the potential use of shrimp by-products for improving the oxidative stability of lard during storage, the composition of the supernatant was not determined. In this context, further research is needed [22].

AX was subsequently incorporated into lard at varying final concentrations, as listed in Table 1. These concentrations were selected based on commercially available AX formulations, particularly soft gel supplements.

Considering the aim of simulating potential commercial applications, inert gas purging of the vials was intentionally omitted [1].

For all samples coded AX 0.25 to AX4, after their preparation, the AX concentration was determined on days 0, 10, and 30 according to the method previously described.

According to Liu et al. [22] and Niizawa et al. [23], the amplitude of the first derivative (dA/dλ) is directly proportional to the concentration of the analyte. The first-order derivative spectrum of each sample revealed two clearly distinguishable peaks. For each peak, both the maximum dA/dλ values and the area under the curve (AUC) were calculated using the method described by [24]:

(1) $${\mathrm{AUC}}_{1}\;=\;\int_{420}^{450}A_{i}d\lambda$$

(2) $${\mathrm{AUC}}_{2}=\int_{455}^{480}A_{j}d\lambda$$

where A is the absorbance at the wavelength λ expressed in nm.

### 2.3. Oxidation Conditions of Lard Samples

The “Schaal oven stability test”—an accelerated storage method that simulates real-time fat oxidation—was employed to investigate the oxidative degradation of lard containing varying concentrations of AX [25,26]. The samples, prepared as previously described, were distributed in shallow dishes to form a uniform layer approximately 0.5 cm thick. They were then stored in the dark at 40 °C under ambient relative humidity (around 50%) for a period of 30 days [27]. The selected temperature was intended to accelerate the thermal–oxidative degradation of lard [25], while minimizing significant AX degradation [15].

### 2.4. UV-Vis Spectral Analysis of AX

The concentration of AX during storage was determined spectrophotometrically using a UV-1900i spectrophotometer (Shimadzu, Kyoto, Japan), within the wavelength range of 350–600 nm and a spectral bandwidth of 0.5 nm [22]. First-order derivative spectra were also recorded using the LabSolutions UV-Vis software (version 1.11).

### 2.5. Differential Scanning Calorimetry (DSC) Analysis

Thermal analysis was performed using an SDT Q600 simultaneous DSC-TGA calorimeter (TA Instruments, New Castle, DE, USA), calibrated with zinc and indium standards. Kinetic analysis was conducted by heating samples of 3.5 ± 0.2 g placed in platinum crucibles at variable heating rates of 5, 7.5, 10, and 15 °C/min, in accordance with the guidelines established by the International Confederation for Thermal Analysis and Calorimetry (ICTAC) [28]. Each sample was initially equilibrated at 35 °C for 1 min under a nitrogen flow of 50 mL/min, followed by continuous heating under an oxygen flow of 50 mL/min up to 300 °C.

The onset temperature of oxidation (To) was determined from the heat flow versus temperature curves as the point where the heat flow signal exhibited a significant deviation from the baseline, indicating the start of the oxidative reaction. Data acquisition and analysis were performed using the Universal Analysis 2000 software (TA Instruments).

The activation energy (Ea) was further determined using the Ozawa–Flynn–Wall (OFW) method, as previously described by Drăghici et al. [29]. Briefly, the OFW method is widely applied in non-isothermal kinetic studies and is based on the following simplified equation:

logβ = log((AEa)/R) − 2.315 − 0.4567 Ea/R × 1/T(3)

where β represents the heating rate (K min^−1^), T is the absolute temperature (K), A is the pre-exponential factor, and R is the universal gas constant.

Equation (3) indicates that β is a function of T:

(4) $$\mathrm{Log}\beta \;=\;a\;\times \;\frac{1}{T}\;+\;b$$

Plotting log β against 1/T, the slope (a) and the constant (b) of the curve can be determined, while the activation energy (Ea) and the pre-exponential factor (A) can be calculated using the following relations:

(5) $$a=-0.4567\frac{E_{a}}{R}$$

(6) $$b=-2.315+log\left(A\frac{E_{a}}{R}\right)$$

### 2.6. Peroxide Value (PV) Assay

The PV measurement was performed using the iodometric standard procedure (Farmacopeea Romana, 1993) [30]. The results were expressed as meq O_2_/kg.

### 2.7. Determination of 2-Thiobarbituric Acid Value (TBA)

The TBA value was determined following the standardized IUPAC method (1989) [31]. In this context, the TBA value represents the increase in absorbance at 530 nm resulting from the reaction between 1 mg of sample (per 1 mL solution) and 2-thiobarbituric acid.

### 2.8. Statistical Analysis

A single batch of shrimp by-products was used in this study and all experiments were conducted in duplicate. Statistical analysis was performed using Statistics Kingdom, applying a one-way analysis of variance (ANOVA) to assess differences between group means at a significance level of *p* < 0.05, followed by Tukey’s post hoc test for multiple comparisons with a 5% significance threshold.

To investigate the combined effects of storage time and AX antioxidant concentration on the parameters T_0_, Eₐ, PV, and TBA, Response Surface Methodology (RSM) was applied. The study considered two independent variables: concentration of AX with 8 different levels (X_1_) and time (X_2_) measured at 3 intervals: 0, 10, and 30 days. The dependent variables analyzed were four responses: T_0_ (Y_1_), Eₐ (Y_2_), PV (Y_3_), and TBA (Y_4_).

A second-order polynomial model was applied for the RSM, including linear, quadratic, and interaction terms between the independent variables, according to the general equation

Y_i_ = β_0_ + β_1_⋅X_1_ + β_2_⋅ X_2_+ β_3_⋅ X_1_^2^ + β_4_⋅ X_2_^2^ + β_5_⋅ X_1_⋅ X_2_ (7)

where Y_i_ represents the dependent variable (T_0_, Eₐ, PV, or TBA), X_1_ and X_2_ are the independent variables (concentration and time), and β is the model coefficient.

As seen in Table 7, the experimental design was structured to cover all levels of the two independent variables, ensuring robust estimation of coefficients and their interactions. The independent variables were coded to facilitate interpretation and comparison of the coefficients.

Mathematical models were implemented via a Python script integrated into the Orange Data Mining (version 3.39.0). Model validation was performed using analysis of variance (ANOVA) and adequacy tests [32]. For the simultaneous optimization of the four responses, a desirability function approach was employed, enabling the identification of the optimal concentration and time conditions for maximizing the performance of the dependent variables.

In addition, Bland–Altman analysis was employed to assess the agreement between the experimental and predicted values obtained from the regression models [33]. These plots were generated using Microsoft Excel.

This approach involves graphically representing the difference between each pair of experimental and predicted values against their mean. In each plot, the mean of the differences (also known as the bias), which indicates the average systematic error between the two sets of values, was shown. Additionally, the limits of agreement (mean ± 1.96 SD) were plotted, within which 95% of the differences are expected to fall, assuming a normal distribution of the differences.

The analysis was performed for the dependent variables To, Ea, PV, and TBA.

## 3. Results and Discussion

From a technological perspective, identifying a method to slow down oxidative processes in lard during storage is of particular interest. The use of astaxanthin extracted from shrimp by-products may represent a promising approach for lard preservation. To properly interpret the experimental results, the initial focus was placed on monitoring the concentration of astaxanthin over time. The findings are especially relevant given that the samples were stored at 40 °C in the dark for a period of 30 days.

### 3.1. Modeling AX Degradation in Lard During Storage

**Figure 2:**
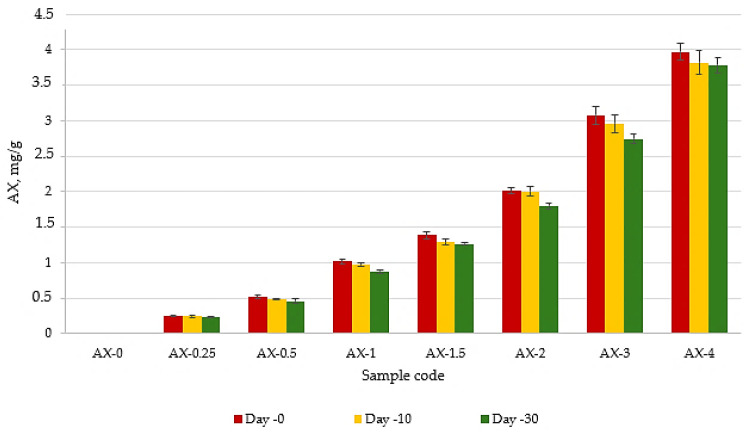
AX concentration dynamics during the storage period.

In the present study, a spectrophotometric method was used to evaluate the thermal stability of the antioxidant pigment astaxanthin (AX) during a 30-day storage period at 40 °C, in darkness, and at a relative humidity of approximately 50%, in lard samples supplemented with this compound. The results are shown in Figure 2. It can be observed that the AX concentration decreases over time by 5–14%. Moreover, a statistically significant difference was observed only for the values recorded on day 30 (*p* < 0.05). A similar decrease in concentration was also observed by Zeng et al. during the storage of Antarctic krill oil, which was attributed to the oxidation of AX [34].

In general, the spectral analysis of AX-enriched samples revealed a broad absorption band in the 400–550 nm region. As previously shown by Casella et al. [15], first-order derivative spectrophotometry can be used to improve spectral resolution. Therefore, the most suitable method to highlight the degradation of AX over time is further investigated.

**Figure 3:**
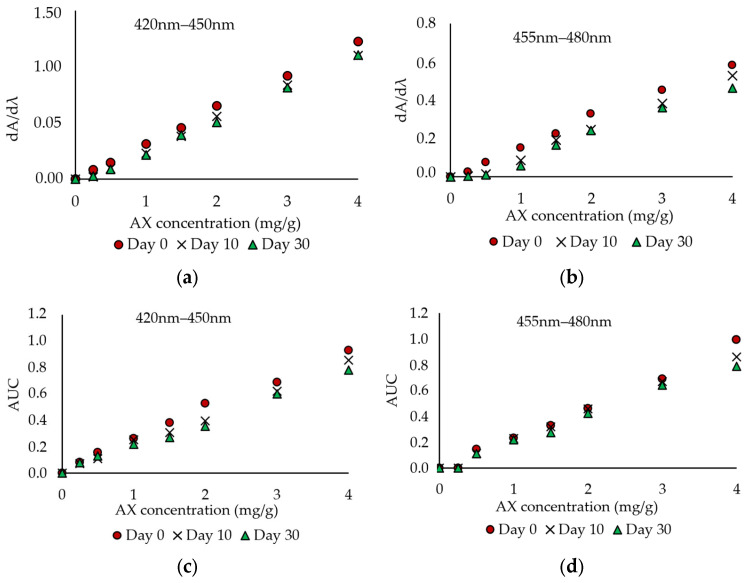
(**a**,**b**) The derivative amplitude dA/dλ values and (**c**,**d**) AUC (area under the curve) for wavelength ranges of 420–450 nm and 455–480 nm.

Figure 3 presents the peak dA/dλ values along with the corresponding AUCs derived from the first-order derivative spectra. As previously observed for various compounds [26], a linear relationship was established between AX concentration and both dA/dλ and AUC values. The regression equations and their respective correlation coefficients (R^2^) are summarized in Table 2, where [AX] represents the AX concentration in the lard samples.

Similar regression equations, with correlation coefficients > 0.99, were previously reported by other authors for decreasing AX concentrations from *Haematococcus pluvialis* or a drug [26].

Based on the data presented in Figure 3 and Table 2, the thermal degradation of AX at 40 °C during storage was confirmed by the progressive decrease in the slopes of the calibration curves. This decrease in AX concentration was especially noticeable when comparing the slopes on day 30 with those on day 0. The most pronounced effect was observed in the regression equations correlating AUC values with AX concentration, particularly within the 420–450 nm wavelength range.

Further research is needed to determine whether future analyses should be based on the spectrophotometrically determined AX concentrations or on the time-dependent AUC values within the 420–450 nm interval. In this study, however, all subsequent analyses will use AX concentrations determined by spectrophotometric methods.

Previous research has demonstrated that astaxanthin (AX) concentration decreases over time, with the rate of degradation being influenced by storage temperature, prior thermal treatments, and exposure to oxygen.

Becerra et al. reported that, after 60 days of sun drying, the AX concentration in shrimp decreased to 74% of its initial value. They also showed that AX degradation follows first-order kinetics, primarily due to the increased accumulation of free astaxanthin released from the hydrolysis of its esterified form during sun drying [35].

Similarly, Li et al. observed a gradual reduction in AX content in shrimp over a 45-day storage period, reaching 80.64% of the initial concentration. This degradation was also associated with noticeable color changes, with shrimp color shifting from reddish-orange to a brownish-yellow hue. The study further highlighted that both AX degradation and color alteration depend on the surrounding matrix. In particular, shrimp oil from *Litopenaeus vannamei*, which is rich in polyunsaturated fatty acids (DHA and EPA), contains unsaturated esters of AX that exhibit lower oxidative stability. Depending on the storage conditions, the degradation of AX may have multiple causes [36].

AX is highly sensitive to oxidative degradation due to the presence of conjugated double bonds in its structure, which can lead to color loss through reactions such as epoxidation and cleavage into low-molecular-weight compounds, including aldehydes and ketones. These oxidized products can significantly impair AX’s biological activity [36].

Furthermore, Qiao et al. suggested that AX degradation can also be enzymatically driven by lipoxygenase and peroxidase, which may be present in the environment or co-extracted with AX from shrimp tissues [37].

As no studies to date have addressed AX extraction using lard as a solvent, further research is needed to clarify the mechanisms underlying the degradation of AX under these specific conditions.

### 3.2. Thermal–Oxidative Stability of Lard Enriched with AX

Thermal analysis is one of the frequently and efficiently applied techniques for evaluating the thermal–oxidative stability of lipid samples [38,39].

The results on the onset temperature of oxidation of lard samples at time zero and after 10 and 30 storage days, as determined by the DSC method, are presented in Table 3.

At day 0, all antioxidant-treated samples exhibited slightly higher oxidation onset temperatures (To) compared to the control sample (AX-0), with closely grouped values around 240–241 °C and no statistically significant differences. The AX-4 sample recorded the highest To (241.19 °C), suggesting a moderate immediate protective effect.

After 10 days of storage, a significant increase in To values was observed for all treated samples, with maximum values reaching 246.86 °C for AX-0.5 and 246.61 °C for both AX-1 and AX-2. In contrast, the control sample showed a notable decrease in To (238.32 °C), indicating advanced lipid oxidation in the absence of antioxidant protection. This stage highlights the peak effectiveness of AX at medium to high concentrations.

By day 30, a general decrease in To values was observed, consistent with progressive oxidative degradation over time. Nonetheless, samples AX-1.5 and AX-2 maintained higher To values than the control (235.78 °C and 235.61 °C vs. 232.46 °C), demonstrating effective residual antioxidant activity. Although AX-4 initially showed strong performance, its long-term stability was reduced, possibly due to saturation effects or diminished antioxidant efficiency at higher concentrations.

Overall, intermediate AX concentrations (AX-1, AX-1.5, and AX-2) provided the best balance between initial efficacy and long-term stability. The control sample, which lacked any antioxidant, exhibited the most rapid decline in thermal stability, underscoring the protective role of AX in delaying oxidative degradation in animal fats.

Previous studies by Wirkowska-Wojdyła et al., reported slightly lower oxidation onset temperatures, which may be attributed to the use of high-pressure DSC analysis [40]. Similar To values were obtained by Marinho et al. in cheese coated with lard and rosemary [41]. Additionally, Islam et al. demonstrated a significant and consistent downward trend in T_0_ values during the storage of oils, with oxidation onset temperatures decreasing over time [42].

The thermal analysis under non-isothermal conditions, with heating at different rates, allows the calculation of the activation energy (Ea), which is the energy required to initiate oxidative processes [40]. The activation energy values obtained by applying the Ozawa–Flynn–Wall method are shown in Table 4.

On day 0, all antioxidant-treated samples exhibited higher activation energy (Ea) values compared to the control AX-0 (91.21 kJ/mol), indicating an immediate protective effect. The Ea of the control sample at day 0 is similar to other reported values on lard, such as 90.3–93.4 kJ/mol [3]. Samples AX-2 (104.62 kJ/mol), AX-3 (101.37 kJ/mol), and AX-4 (106.07 kJ/mol) showed the highest initial values, suggesting strong antioxidant efficiency in thermally stabilizing the system. Lower concentrations (AX-0.25 and AX-0.5) also presented elevated Ea values, albeit slightly lower, while AX-1 and AX-1.5 displayed intermediate levels (–100 kJ/mol).

After 10 days of storage at 40 °C, divergent trends were observed. Lower-concentration samples (AX-0.25, AX-0.5) and the control showed a significant decrease in Ea (85–86 kJ/mol), indicating accelerated degradation and loss of antioxidant activity. In contrast, AX-2, AX-3, and AX-4 displayed an increase in Ea (up to 102.96–108.72 kJ/mol), suggesting either delayed antioxidant activation or prolonged effectiveness under thermal stress. Notably, AX-4 reached the highest recorded value (108.72 kJ/mol) on day 10, which may imply a slow release mechanism or a potential antioxidant regeneration effect.

By day 30, most treated samples maintained Ea values above 100 kJ/mol, with minimal variations. AX-0.5, AX-1, AX-1.5, and AX-2 demonstrated good stability, confirming the persistence of the protective effect over time. AX-4 showed a slight decrease (to 99.48 kJ/mol), yet still remained within an effective range.

This progression underscores the concentration- and time-dependent efficacy of AX. Medium concentrations (AX-1 to AX-2) provided an optimal balance between immediate antioxidant activity and long-term stability, consistent with prior findings from oxidation onset temperature analysis. Conversely, lower concentrations (AX-0.25, AX-0.5) rapidly lost efficacy, while higher concentrations (AX-4), although initially potent, may exhibit long-term instability—possibly due to pro-oxidant effects at elevated doses.

The presence of antioxidants within a lipid matrix plays a pivotal role in mitigating oxidative degradation. By interfering with the free radical chain reactions that drive lipid oxidation, antioxidants effectively inhibit or delay the formation of primary and secondary oxidation products. This interference alters the reaction kinetics, often manifested as an increase in the activation energy (Ea) required for the oxidative process to proceed [43]. A higher Ea suggests that more energy is necessary to initiate the degradation reaction, reflecting the enhanced oxidative stability of the system.

In support of this, Wirkowska-Wojdyła et al. reported elevated Ea values in systems containing enzymatically interesterified blends of lard, rapeseed oil, and concentrated fish oil (at a weight ratio of 7:2:1) subjected to high-pressure oxidation conditions [40]. This formulation likely benefited from synergistic antioxidant effects derived from the unsaturated fatty acid content and natural bioactive compounds present in the fish oil concentrate.

Conversely, Wang et al. observed substantially lower activation energy values in the context of lard transesterification for biodiesel production. The reduction in Ea in that case may be attributed to the catalytic and thermal conditions specific to fuel synthesis, which favor rapid molecular breakdown rather than stabilization. These contrasting results highlight the significance of formulation type, processing conditions, and the functional role of antioxidants in modulating oxidative kinetics.

### 3.3. The Chemical Study of the Oxidation Process

Lipid oxidation typically progresses through two distinct phases: (1) the initial formation of hydroperoxides and (2) the subsequent generation of malondialdehyde compounds [44]. In the present study, these stages were assessed in lard samples through peroxide value and thiobarbituric acid (TBA) assays

The peroxide value (PV), expressed in meq O_2_/kg fat, reflects the extent of primary peroxide formation—initial products of lipid oxidation. As such, PV serves as a sensitive indicator of the early oxidative state and the progression of the oxidation process over time.

As shown in Table 5, at day 0, all samples exhibited low PVs (2.30–2.64), with no significant differences between the antioxidant-treated groups and the control. This suggests that the system was initially stable, with oxidative reactions only beginning to occur. Slightly elevated PVs in AX-2 and AX-3 (2.57 and 2.64, respectively) may reflect minor differences in system homogenization or subtle interactions between AX compounds and lipid components.

After 10 days of storage, PVs increased in all samples, indicating ongoing oxidative progression. The control (AX-0) and AX-0.25 both reached a value of 6.71, suggesting that this minimal concentration provided little to no protection. In contrast, AX-1, AX-1.5, AX-2, and AX-3 demonstrated more effective inhibition of peroxide formation (ranging from 5.88 to 6.57), with AX-1.5 exhibiting the lowest PV (5.88), followed closely by AX-4 (5.39), indicating a strong antioxidant effect at this stage.

By day 30, PVs increased substantially across all samples, yet inter-treatment differences became more pronounced. The control group exhibited the highest PV (13.86 meq O_2_/kg), indicating advanced oxidation in the absence of antioxidant protection. AX-4 (11.97), AX-2 (12.08), AX-3 (12.15), and AX-1 (12.22) showed more effective peroxide suppression, suggesting sustained antioxidant activity over time. Lower concentrations (AX-0.25 and AX-0.5) proved less effective, with values approaching that of the control, while AX-1.5, despite its efficacy on day 10, increased significantly to 13.48, suggesting depletion of antioxidant capacity over prolonged storage.

These results suggest that intermediate to high concentrations of AX—particularly AX-1, AX-2, and AX-4—provide meaningful protection against peroxide formation over time, whereas lower concentrations (AX-0.25 and AX-0.5) fail to efficiently inhibit lipid oxidation. PV data thus reinforce prior observations and support the hypothesis that moderate to high doses of AX are most effective in preventing primary oxidation during high-temperature storage.

Pop and Dippong reported that peroxide values were consistently lower in lard compared to goose fat, suggesting a higher oxidative stability of lard under identical conditions [45]. Moreover, the addition of burdock extract significantly reduced PV in both fat types, demonstrating its antioxidant potential across different lipid matrices. Similarly, Pu et al. investigated the use of AX in flaxseed oil and observed a notable reduction in primary oxidation products [18]. The enhanced efficacy of AX in this case was attributed to the oil’s inherently high content of polyunsaturated fatty acids, which are more susceptible to oxidative degradation and therefore more responsive to antioxidant intervention. These findings collectively underscore the importance of both fat composition and antioxidant compatibility in determining oxidative resistance during storage.

The TBA assay is a widely used indicator of secondary oxidation products, particularly malondialdehyde (MDA), which accumulates during the advanced stages of lipid oxidation. Table 6 presents the TBA values measured in lard samples treated with varying concentrations of AX across three storage intervals (0, 10, and 30 days). The results show a consistent increase in TBA content over time in all sample groups, indicating the progressive nature of lipid oxidation. The control sample (AX-0) consistently exhibited the highest TBA values at each time point, reaching a maximum on day 30, thereby confirming the absence of antioxidant protection.

The incorporation of AX into lard samples led to a clear reduction in lipid oxidation, as evidenced by lower TBA values compared to the untreated control. The antioxidant effect intensified with increasing AX concentration; however, this effect plateaued from AX-1.5 onward, suggesting the presence of an efficiency threshold beyond which further supplementation yields diminishing returns. Overall, the data support the efficacy of AX in mitigating lipid oxidation in lard. AX-1.5 and AX-2 appear to offer an optimal balance between antioxidant activity and practical resource use.

Interestingly, higher AX concentrations (notably AX-4) resulted in slightly elevated TBA levels, which may be attributed to the biphasic behavior of some antioxidants, where excessive dosing can induce pro-oxidant effects under specific conditions.

Abdelmalek et al., investigated the antioxidant efficacy of AX in comparison to BHA (butylated hydroxyanisole) in marinated chicken meat. Their findings revealed comparable TBARS values in samples treated with AX and those treated with BHA, suggesting that AX performs on par with conventional synthetic antioxidants in retarding lipid oxidation [46].

Moreover, studies on the oxidative behavior of lard under high-temperature storage conditions demonstrated that TBA index progression at 50 °C closely follows an exponential trend over time, reinforcing the time-dependent acceleration of secondary oxidation under thermal stress [47].

### 3.4. Optimization of AX Concentration and Temporal Evaluation of Its Antioxidant Effect Through Response Surface Methodology (RSM)

A systematic approach that enables a comprehensive interpretation of AX’s efficacy in preventing lipid oxidation over time is the use of multiple linear regression with interaction terms. This model allows for the evaluation of the combined influence of AX concentration and storage time on key parameters such as oxidation onset temperature (To), activation energy (Ea), peroxide value (PV), and TBA values (Table 7).

**Table 7:** Response Surface Methodology.

	Independent Variables		Dependent Variables			
No	Concentration in AX (mg /g)	Time	To	Ea (kJ/mol)	PV (meq O_2_/kg)	TBA Value
	X_1_	X_2_	Y_1_	Y_2_	Y_3_	Y_4_
1	0	0	239.48	91.21	2.42	0.0210
2	0.25	0	238.93	100.46	2.30	0.0191
3	0.52	0	239.38	99.97	2.46	0.0191
4	1.01	0	240.75	100.47	2.33	0.0202
5	1.48	0	240.03	98.90	2.35	0.0270
6	2.01	0	240.85	104.62	2.57	0.0307
7	3.07	0	240.93	101.37	2.64	0.0350
8	3.97	0	241.19	106.07	2.39	0.0368
9	0	10	238.32	86.89	6.71	0.0245
10	0.24	10	245.82	86.30	6.71	0.0305
11	0.48	10	246.86	85.43	7.34	0.0310
12	0.97	10	246.61	94.59	6.38	0.0289
13	1.29	10	246.03	95.01	5.88	0.0344
14	1.97	10	246.61	102.96	6.57	0.0372
15	2.96	10	245.83	100.65	6.48	0.0418
16	3.82	10	244.58	108.72	5.39	0.0396
17	0	30	232.46	94.57	13.86	0.0439
18	0.23	30	234.83	100.39	13.62	0.0446
19	0.45	30	239.77	101.56	13.41	0.0422
20	0.87	30	239.08	101.48	12.22	0.0419
21	1.27	30	235.78	101.38	13.48	0.0434
22	1.80	30	235.61	101.20	12.08	0.0456
23	2.74	30	234.96	100.42	12.15	0.0449
24	3.78	30	234.64	99.48	11.97	0.0460

The regression coefficients of the model and the analysis of variance (ANOVA) results are presented in Table 8. Following the ANOVA test, the model was recalculated by removing the non-significant terms at a significance level of *p* > 0.05. The model was evaluated based on R^2^, adjusted R^2^, and predicted R^2^, which assess how well the model explains and predicts the data. The standard error indicates the accuracy of the estimates, while the F-statistic and *p*-value reflect the significance of the relationships within the model. The Lack of Fit test and its corresponding *p*-value assess whether the model fits the data adequately, indicating whether further improvements are needed.

Table 8 summarizes the regression coefficients, significance levels, and model diagnostics for the responses: To, Ea, PV, and TBA value. The influence of the independent variables AX concentration and time was evaluated through linear, quadratic, and interaction terms, with significance determined at *p* < 0.05.

For the To response, the model displayed a strong fit (R^2^ = 0.8315; adjusted R^2^ = 0.8238; predicted R^2^ = 0.5903). Both AX concentration (β_1_, *p* = 0.0201) and time (β_2_, *p* = 0.0002) had a significant linear effect. The quadratic terms for AX concentration (β_3_, *p* = 0.0403) and time (β_4_, *p* = 0.0001) were also significant, indicating nonlinear relationships. The AX concentration × time interaction (β_5_, *p* = 0.2902) was not statistically significant. The Lack of Fit test (*p* = 0.1117) showed no significant deviation, confirming that the model adequately fits the data.

For the Ea response, the model showed moderate predictive power (R^2^ = 0.6682; adjusted R^2^ = 0.6532; predicted R^2^ = 0.3920). The linear term for AX concentration was significant (β_1_, *p* = 0.0044), while time (β_2_, *p* = 0.0362) was also close to the significance threshold. The quadratic terms for AX concentration were not significant. The model fit was acceptable as indicated by the Lack of Fit test (*p* = 0.7497).

In the case of PV, the model performed exceptionally well (R^2^ = 0.9929; adjusted R^2^ = 0.9925; predicted R^2^ = 0.9746). Among all terms, only the AX concentration × time interaction (β_5_, *p* = 0.0107) had a statistically significant influence, highlighting the importance of the combined effect of the two factors. The Lack of Fit test (*p* = 0.9045) further confirmed the model’s adequacy.

For the TBA value, the model exhibited high predictive accuracy (R^2^ = 0.9368, Adjusted R^2^ = 0.9339, Predicted R^2^ = 0.7558). Significant effects were observed for both AX concentration (β_1_, *p* = 0.0002) and time (β_2_, *p* = 0.0001), as well as for the interaction term AX concentration × time (β_5_, *p* = 0.0001), suggesting both individual and combined influences of the factors. Quadratic effects were not significant. The Lack of Fit test (*p* = 0.3962) indicated no need for model adjustments.

Overall, the statistical indicators, high F values, low model *p*-values (*p* < 0.001), and low standard errors support the validity and precision of the models. The non-significant Lack of Fit tests across all responses confirm that the models appropriately describe the experimental data, without the necessity for additional complexity.

**Figure 4:**
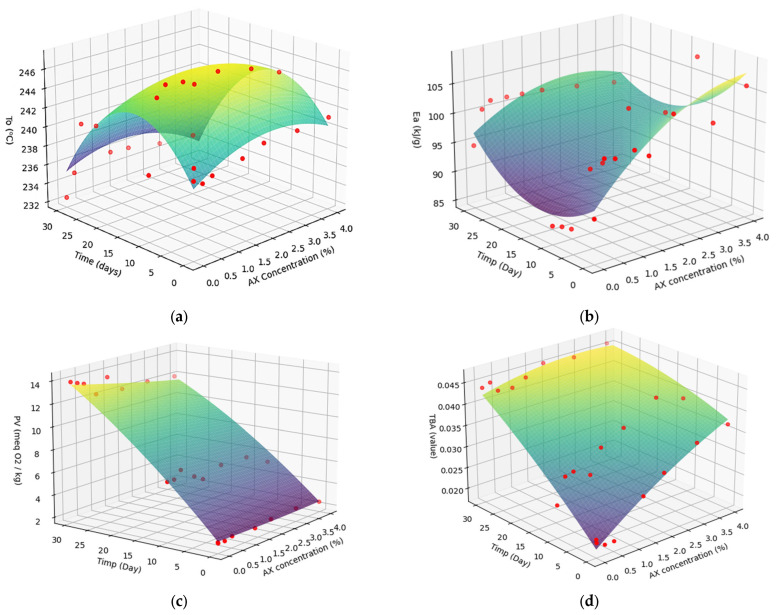
Response Surface Methodology (RSM) graph illustrating the interactive effects of AX concentration and time on T_0_ (**a**), Eₐ (**b**), PV (**c**), and TBARS (**d**), with the red dots representing the experimentally determined values.

The simultaneous effects of AX concentration and time on these variables are illustrated in Figure 4.

The three-dimensional response surface plots (Figure 3) illustrate the influence of the independent variables—AX concentration and storage time—on the dependent variables: onset temperature (To), activation energy (Ea), peroxide value (PV), and TBA value.

As shown in Figure 3a, To increases with AX concentration up to approximately 2 mg/g, after which it begins to decline. This pattern suggests that moderate AX concentrations are effective in enhancing oxidative stability by delaying the onset of lipid degradation. Similarly, Ea exhibits a general increasing trend with AX concentration, particularly beyond 2.5–3 mg/g, indicating an improvement in thermal resistance. However, at early storage times and lower concentrations, the Ea values show greater variability, likely due to insufficient antioxidant protection under these conditions.

PV, an indicator of primary lipid oxidation, decreases significantly with increasing AX concentration, particularly above 2 mg/g, reaching minimum values at 3–4 mg/g. This demonstrates the strong inhibitory effect of AX on hydroperoxide formation at higher concentrations. Likewise, the TBA value, which reflects secondary oxidation products such as malondialdehyde, decreases as AX concentration increases, with the lowest values recorded around 3–3.5 mg/g. This trend further confirms the antioxidant efficacy of AX in mitigating lipid oxidation.

The protective effect of AX at concentrations up to 2 mg/g oil has also been demonstrated in flaxseed oil, based on reductions in PV and TBA value [48].

The observed decline in To and Ea at higher AX concentrations may be attributed to the increased co-extraction of unsaturated lipids along with AX from shrimp by-products. This hypothesis is supported by El-Bialy and Abd El-Khalek, who reported elevated levels of polyunsaturated fatty acids in AX extracts obtained from shrimp waste through lactic fermentation combined with vegetable oil extraction [49]. In contrast, Pu et al. found that AX extraction using flaxseed oil did not significantly alter the linoleic acid or other polyunsaturated fatty acid content of the carrier oil. These findings suggest that the lipid composition of the extraction solvent may influence the final oxidative stability of AX-enriched systems [37]. Future studies should investigate the compositional changes associated with AX extraction using different lipid matrices, including lard.

Considering all measured responses, the optimal AX concentration appears to lie within the range of 2.5–3.5 mg/g, where To and Ea are maximized, and PV and TBA values are minimized. This concentration range offers a balance between enhanced oxidative protection and thermal stability, making approximately 3 mg/g AX a suitable choice from both a technological and preservative standpoint.

To illustrate the prediction performance of these models, four Bland–Altman plots were generated.

Bland–Altman analysis is a statistical method for evaluating the agreement between two quantitative measurement techniques by defining limits of agreement. The corresponding plot facilitates the identification of systematic bias and highlights potential outliers or inconsistencies between measurements.

**Figure 5:**
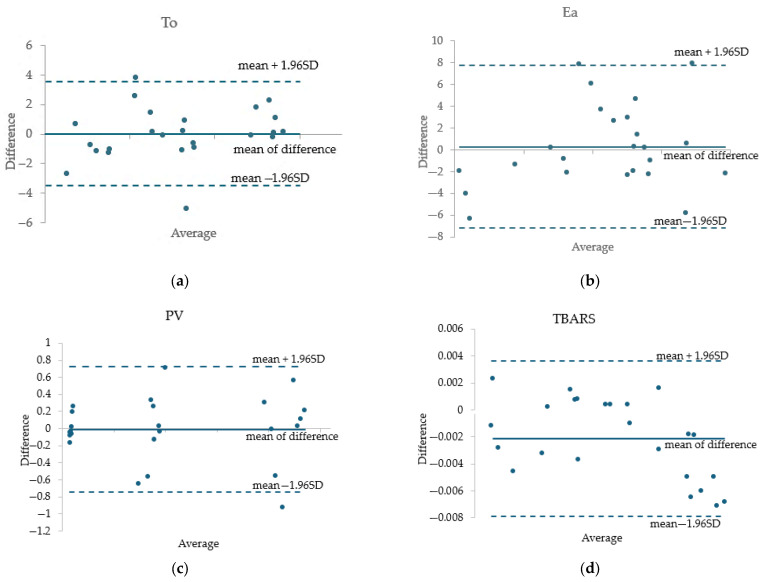
Bland–Altman Plots for the validation of predicted vs. experimental values of To (**a**), Ea (**b**), PV (**c**), and TBARS (**d**).

To validate the regression models developed for To, Ea, PV, and TBA, Bland–Altman plots were constructed (Figure 5) by comparing the experimental values with those predicted by the models. The analysis of these plots demonstrated good agreement between the two datasets. Most values fell within the limits of agreement (mean ± 1.96 SD), indicating no substantial systematic bias. For To and PV, the mean of the differences had values close to zero (0.0083 and −0.0085, respectively), confirming high model accuracy. Ea showed slightly increased variability without consistent over- or underestimation. However, the mean of the differences was equal to 0.2833, but it is very small compared to the experimental values obtained for the activation energy. TBA exhibited a mild underestimation trend, with a very small mean systematic difference (−0.0021), which suggests excellent agreement between the experimental and calculated values.

Overall, the Bland–Altman analysis supports the validity and robustness of the regression models in describing the oxidative behavior of the system.

## 4. Conclusions

This study is the first to investigate the influence of astaxanthin (AX), extracted from shrimp by-products, on lard during storage. A simple, eco-friendly, and cost-effective extraction method was proposed. The results showed that after 30 days of storage under oxidative stress conditions (40 °C, in the dark), a significant degradation of AX was observed across all lard samples, with up to 86% reduction from the initial concentration. This degradation may be attributed to several mechanisms, including oxidation due to the presence of conjugated double bonds in the AX structure, as well as potential enzymatic activity, such as lipoxygenase or peroxidase. In addition to spectrophotometric quantification, spectral analysis, specifically the measurement of the area under the curve in the 420–450 nm range, may offer a more precise method for tracking AX concentration over time. Further research is needed to better correlate and validate these analytical approaches.

Moreover, AX was found to delay both the initiation and propagation of lipid oxidation, as evidenced by increases in oxidation onset temperature (To) and activation energy (Ea), along with reductions in peroxide value (PV) and TBARS. Optimization using Response Surface Methodology (RSM) identified –3 mg/g AX as the most effective concentration, providing a balanced compromise between thermal stability and oxidative protection. Additionally, the color of lard was more strongly influenced by the presence of AX than by its specific concentration, but the color has a pleasant appearance. Future research should focus on identifying and quantifying bioactive compounds in shrimp by-products, monitoring their stability over time, and evaluating the mechanisms by which they provide oxidative protection to fats.

## Figures and Tables

**Table 1:** The ratio of AX extract/lard, the sample concentration of AX (mg/g), and the color of the samples expressed in the CIELAB space.

Sample	AX-0	AX-0.25	AX-0.5	AX-1	AX-1.5	AX-2	AX-3	AX-4
Ratio								
Supernatant with 4 mg/g AX	0.000	0.250	0.500	0.625	0.750	0.875	0.938	1.000
lard	1.000	0.750	0.500	0.375	0.250	0.125	0.063	0.000
Sample concentration in AX, mg/g	0	0.25	0.5	1	1.5	2	3	4
L*	85.91	84.38	83.95	83.47	83.38	81.32	76.34	72.38
a*	0.58	10.78	11.26	13.21	14.62	16.58	17.61	27.55
b*	1.44	51.34	51.56	58.23	60.4	58.42	57.27	55.49

**Table 2:** Comparison of linear regression equations of the petroleum ether extracts of AX obtained from the samples stored in the dark at 40 °C, at different wavelength ranges.

Field	Day	Equation	R^2^
The range of wavelengths 420–450 nm			
	0	dA/dλ = 0.0307 × [AX] + 0.0005	0.9988
dA/dλ	10	dA/dλ = 0.0287 × [AX] − 0.0036	0.9973
	30	dA/dλ = 0.0283 × [AX] − 0.004	0.9971
	0	AUC = 0.2278 × [AX] + 0.0302	0.9947
AUC	10	AUC = 0.2070 × [AX] + 0.0098	0.9953
	30	AUC = 0.1895 × [AX] + 0.0137	0.9927
The range of wavelengths 455–480 nm			
	0	dA/dλ = 0.1493 × [AX] + 0.0012	0.9958
dA/dλ	10	dA/dλ = 0.1379 × [AX] − 0.0293	0.9919
	30	dA/dλ = 0.1253 × [AX] − 0.0290	0.9842
	0	AUC = 0.2459 × [AX] − 0.0199	0.9928
AUC	10	AUC = 0.2232 × [AX] − 0.0107	0.9950
	30	AUC = 0.2065 × [AX] − 0.0093	0.9909

[AX] is the AX concentration in the investigated lard and R^2^ is the correlation coefficient.

**Table 3:** The onset temperatures of lard oxidation, obtained from the DSC curves of samples heated at 10 °C/min.

Samples	Onset Temperatures of Lard Oxidation (°C)		
	Day 0	Day 10	Day 30
AX-0	239.48 ^b^ ± 0.35	238.32 ^a,b^ ± 0.94	232.46 ^a^ ± 0.22
AX-0.25	238.93 ^a,b^ ± 0.56	245.82 ^b^ ± 0.23	234.83 ^a^ ± 0.65
AX-0.5	239.38 ^a,b^ ± 0.06	246.86 ^b^ ± 0.36	239.77 ^a^ ± 0.53
AX-1	240.75 ^a,b^ ± 0.30	246.61 ^b^ ± 0.12	239.08 ^a^ ± 0.69
AX-1.5	240.03 ^a,b^ ± 0.91	246.03 ^b^ ± 0.93	235.78 ^a^ ± 0.61
AX-2	240.85 ^a,b^ ±0.01	246.61 ^b^ ± 0.07	235.61 ^a^ ± 0.81
AX-3	240.93 ^a,b^ ± 0.02	245.83 ^b^ ± 0.22	234.96 ^a^ ± 0.87
AX-4	241.19 ^a,b^ ± 0.19	244.58 ^b^ ± 0.38	234.64 ^a^ ± 0.61

Results in the same row followed by the same letters are not significantly different (*p* < 0.05).

**Table 4:** The activation energy (kJ/mol) of the oxidative degradation of lard samples enriched with different concentrations of AX, at storage days 0, 10 and 30.

Samples	Activation Energy (kJ/mol)		
	Day 0	Day 10	Day 30
AX-0	91.21 ^a^ ± 1.89	86.89 ^a^ ± 3.33	94.57 ^a^ ± 2.26
AX-0.25	100.46 ^b^ ± 1.58	86.30 ^a^ ± 3.63	100.39 ^b^ ± 1.97
AX-0.5	99.97 ^b^ ± 2.15	85.43 ^a^ ± 1.81	101.56 ^b^ ± 4.46
AX-1	100.47 ^b^ ± 4.87	94.59 ^b^ ± 5.91	101.48 ^b^ ± 4.72
AX-1.5	98.90 ^b^ ± 1.79	95.01 ^b^ ± 3.34	101.38 ^b^ ± 3.63
AX-2	104.62 ^c^ ± 5.24	102.96 ^c^ ± 4.21	101.20 ^b^ ± 3.67
AX-3	101.37 ^b^ ± 1.66	100.65 ^c^ ± 2.57	100.42 ^b^ ± 2.97
AX-4	106.07 ^d^ ± 3.10	108.72 ^d^ ± 5.95	99.48 ^c^ ± 4.83

Results in the same column followed by the same letters are not significantly different (*p* < 0.05).

**Table 5:** Evolution of the peroxide value (meq O_2_/kg) of lard enriched with AX at different concentrations, over a 30-day period of storage at 40 °C.

Samples	Peroxide Value (meq O_2_/kg)		
	Day 0	Day 10	Day 30
AX-0	2.42 ^a^ ± 0.4	6.71 ^a^ ± 0.1	13.86 ^a^ ± 0.5
AX-0.25	2.3 ^a^ ± 0.2	6.71 ^a^ ± 0.8	13.62 ^a^ ± 1.4
AX-0.5	2.46 ^a^ ± 0.4	7.34 ^b^ ± 0.4	13.41 ^a^ ± 0.3
AX-1	2.33 ^a^ ± 0.1	6.38 ^a^ ± 0.6	12.22 ^b^ ± 0.9
AX-1.5	2.35 ^a^ ± 0.2	5.88 ^c^ ± 0.1	13.48 ^a^ ± 0.9
AX-2	2.57 ^a^ ± 0.4	6.57 ^a^ ± 0.4	12.08 ^b^ ± 1.4
AX-3	2.64 ^a^ ± 0.2	6.48 ^a^ ± 0.5	12.15 ^b^ ± 0.4
AX-4	2.39 ^a^ ± 0.5	5.39 ^d^ ± 0.2	11.97 ^c^ ± 0.1

Results in the same column followed by the same letters are not significantly different (*p* < 0.05).

**Table 6:** Evolution of the TBA value of lard enriched with AX at different concentrations, over a 30-day period of storage at 40 °C.

Samples	TBA Value		
	Day 0	Day 10	Day 30
AX-0	0.33 ^a^ ± 0.04	1.10 ^a^ ± 0.04	3.45 ^a^ ± 0.22
AX-0.25	0.31 ^a^ ± 0.03	1.07 ^b^ ± 0.36	3.36 ^b^ ± 0.5
AX-0.5	0.31 ^a^ ± 0.04	1.05 ^b^ ± 0.40	3.36 ^b^ ± 0.02
AX-1	0.33 ^a^ ± 0.03	1.06 ^b^ ± 0.02	3.31 ^b^ ± 0.26
AX-1.5	0.39 ^b^ ± 0.03	1.05 ^b^ ± 0.41	3.31 ^b^ ± 0.43
AX-2	0.43 ^c^ ± 0.05	1.03 ^c^ ± 0.23	3.32 ^b^ ± 0.23
AX-3	0.47 ^d^ ± 0.11	1.02 ^c^ ± 0.21	3.28 ^c^ ± 0.27
AX-4	0.49 ^d^ ± 0.03	1.02 ^c^ ± 0.20	3.28 ^c^ ± 0.13

Results in the same column followed by the same letters are not significantly different (*p* < 0.05).

**Table 8:** The regression coefficients, significance levels, and model diagnostics for the responses: To, Ea, PV, and TBA value.

Regression Coefficients	To		Ea (kJ/mol)		PV (meq O_2_/kg)		TBA Value	
	Value	*p*	Value	*p*	Value	*p*	Value	*p*
β_0_	238.0207	0.0000	92.5461	0.0000	2.4864	0.0000	0.0187	0.0000
Linear								
β_1_	3.1688	0.0201	7.5508	0.004	−0.0920	0.6053	0.0064	0.0002
β_2_	0.8542	0.0002	−0.6282	0.036	0.4529	0.0002	0.0009	0.0001
Quadratic								
β_3_	−0.6562	0.0403	−0.9080	0.1305	0.0191	0.6960	−0.0004	0.0811
β_4_	−0.0317	0.0001	0.0255	0.0074	−0.0027	0.0111	0.0000	0.1685
Crossed								
β_5_	−0.0333	0.2902	−0.0954	0.0480	−0.0168	0.0107	−0.0001	0.0003
R^2^	0.8315		0.66823853		0.992878016		0.936811529	
Adjusted R^2^	0.8238		0.653158463		0.99255429		0.933939325	
Predicted R^2^	0.5903		0.3920		0.9746		0.7558	
Standard Error	1.6768		2.899064534		0.381263388		0.00274618	
F	108.5671		44.31270356		306.7027083		326.1647769	
*p*	*p* < 0.001				*p* < 0.001		*p* < 0.001	
Lack of Fit	49.4221		0.7176		1.6743		1.0430	
*p* Lack of Fit	0.1117		0.74897		0.9045		0.3962	

## Data Availability

The original contributions presented in the study are included in the article.
